# Cognitive and socio-environmental correlates of condom use among internal migrants in Shenzhen, China: a structural equation model approach

**DOI:** 10.1186/1471-2334-14-S2-P13

**Published:** 2014-05-23

**Authors:** Xiaona Liu, Tan Jingguang, Vicki Erasmus, Lenneke van Genugten, Xinying Sun, Jan Hendrik Richardus

**Affiliations:** 1 Erasmus MC, University Medical Center of Rotterdam, Rotterdam, the Netherlands

## Introduction

Internal migrants have been repeatedly characterized as “the tipping point” for HIV epidemic in China. Despite increasing awareness that condom use of the internal migrants is essential in the battle for reducing the spread of HIV/AIDS in China, little is known as to what factors influencing actual condom use in the population. This study aims to explore cognitive and socio-environmental correlates of condom use comprehensively, and test a model for illustrating condom use among internal migrants in Shenzhen, China.

## Materials and methods

A cross-sectional survey was conducted among a purposive sample of 400 migrants between April 2013 and June 2013. Participants were asked by a questionnaire about their socioeconomic status, HIV-related knowledge, sexual behavior, cognition of condom use, and socio-environmental conditions. A hypothesized model was expanded from constructs of the theory of planned behavior and protection motivation theory to environmental conditions. We performed multivariate analyses and structural equation modeling using SPSS 21.0 and Amos 22.0 to assess the model on explaining condom use.

## Results

Of 268 sexual active participants, 66.8% reported use condom(s) inconsistently in the preceding year, and 51.1% reported did not use condom in their last intercourse. Both measurements of condom use correlated with education, knowledge, cognition (severity, susceptibility, fear, response efficacy, self-efficacy, barriers to action, and subjective norms), environmental conditions (living with a committed partner, living in a community where condoms are easily available, involving in HIV prevention program, discussion on condom with partner) (P<0.05). The hypothesized model used in this study was well fit in illustrating condom use (P=0.035, GFI=0.952, AGFI=0.962, CFI=0.983, NFI=0.997, RMSEA=0.068). Barriers to action were the strongest correlates of consistent condom use (coefficient=0.38, P=0.002), while discussing condom with sexual partner was the strongest correlates of condom use in the latest intercourse (coefficient=0.26, P<0.001).

## Conclusions

Our findings confirmed the model integrating cognitive and socio-environmental factors as a suitable model for Chinese internal migrants regarding condom use. HIV prevention programs may benefit by focusing on barriers to behavior change and environmental conditions for internal migrants in China.

